# Identification of a novel *FBN1* gene mutation in a Chinese family with Marfan syndrome

**Published:** 2011-09-17

**Authors:** Bo Meng, Hongyi Li, Tao Yang, Shangzhi Huang, Xian Sun, Huiping Yuan

**Affiliations:** 1 Department of Ophthalmology, the 2nd Affiliated Hospital of Harbin Medical University, Harbin, China; 2 Department of Medical Genetics, Institute of Basic Medical Sciences, Chinese Academy of Medical Sciences & Peking Union Medical College, WHO Collaborating Centre for Community Control of Hereditary Diseases , Beijing, China; 3 Department of Oncology, the 3rd Affiliated Hospital of Harbin Medical University, Harbin, China

## Abstract

**Purpose:**

To identify the mutation in the fibrillin-1 gene (*FBN1*) in a Chinese family with Marfan syndrome (MFS).

**Methods:**

Patients and family members were given complete physical, ophthalmic, and cardiovascular examinations. Genomic DNA was extracted from leukocytes of venous blood of six individuals in the family and 170 healthy Chinese individuals. All of the 65 coding exons and their flanking intronic boundaries of *FBN1* were amplified in the proband by polymerase chain reaction and followed by direct sequencing. The mutation identified in the proband was screened in the other family members and the 170 healthy Chinese individuals by direct sequencing. Protein conservation analysis was performed in six species using an online ClustalW tool. Protein structure was modeled based on the Protein data bank and mutated in DeepView v4.0.1 to predict the functional consequences of the mutation.

**Results:**

A novel heterozygous c.3703T>C change in exon 29 of *FBN1* was detected in the proband, which resulted in the substitution of serine by proline at codon 1235 (p.S1235P). This mutation was also present in two family members but absent in the other, unaffected family members and the 170 healthy Chinese individuals. The mutant residue located in the calcium binding epidermal growth factor-like#15 domain is highly conserved among mammalian species and could probably induce conformation change of the domain.

**Conclusions:**

We indentified a novel p.S1235P mutation in FBN1, which is the causative mutation for MFS in this family. Our result expands the mutation spectrum of *FBN1* and contributes to the study of the molecular pathogenesis of Marfan syndrome.

## Introduction

Marfan syndrome (MFS, OMIM 154700) is a relatively common autosomal dominant hereditary disorder of the connective tissue, with prominent manifestations in the skeletal, ocular, and cardiovascular systems [1]. It is clinically diagnosed according to the Ghent criteria [2].

MFS is mainly caused by mutations in the human fibrillin-1 (*FBN1*) gene [3]. *FBN1* contains 65 exons encoding a secreted 350 kDa glycoprotein, which is highly conserved among different species [4]. Fibrillin-1 is characterized by a specific module organization in the extracellular matrix and is mainly composed of three types of repeated modules: 47 epidermal growth factor (EGF)-like modules (43 calcium binding [or cb] EGF-like modules and 4 non-cb EGF-like modules), 7 transforming growth factor-binding (or TB) protein-like modules (8 Cys/TB), and 2 hybrid modules [5]. To date, more than 60% of the reported mutations of *FBN1* are missense mutations, and the majority of these are located in the cb EGF modules (in particular, cysteine residues, implicated in disulfide bond formation, or other residues implicated in calcium-binding and/or intra-/intermolecular interactions) [6,7]. There is limited evidence of genotype-phenotype correlations of this disorder, while mutations associated with neonatal MFS and other severe forms have been shown to cluster in exons 24–32 [8-11]. Molecular analysis of *FBN1* is necessary because such analysis can detect at-risk individuals at an early stage and offer the possibility of prenatal diagnosis.

We came across a four-generation family affected with MFS in Northeast China and detected a novel heterozygous mutation in *FBN1.* The mutation was found in the affected individuals but was not observed in any of the healthy family members.

## Methods

### Patients and clinical data

**Figure 1:**
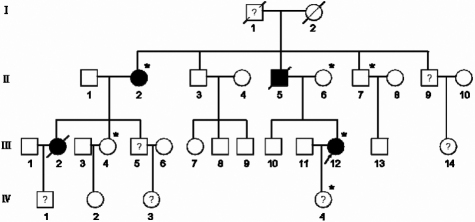
The pedigree of the family. Squares and circles indicate males and females, respectively, and the darkened symbols represent the affected members. Slashed symbols denote that the subject is deceased. Symbols with a question mark in the center indicate that the member is not diagnosed clearly. Asterisks indicate the subjects participating in this study. The patient indicated by the arrow is the proband.

The proband, a 32-year-old female, was diagnosed with MFS according to the Ghent criteria [2]. The family history revealed four affected members over four generations, two of whom were deceased. Available individuals II:2, II:6, II:7, III:4, III:12, and IV:4 were given complete physical, ophthalmic, and cardiovascular examinations after obtaining informed consent (Figure 1). One hundred Chinese controls without diagnostic features of MFS were also recruited. The study was approved by the Harbin Medical University Ethics Committee (Harbin, China).

### Genomic DNA preparation

Whole blood from six members of the family (II:2, II:6, II:7, III:4, III:12, and IV:4) and 170 unrelated Chinese controls were collected in tubes containing EDTA as an anticoagulant. Genomic DNA was extracted using a TIANamp Blood DNA Kit (Tiangen Biltech Co. Ltd, Beijing, China) according to the manufacturer’s protocol.

### Mutation screening

All 65 coding exons and flanking intronic regions, including splice sites of *FBN1,* were amplified by polymerase chain reaction (PCR) using a set of primers listed in Appendix 1. PCR was performed for 35 cycles, with a denaturing phase of 30 s at 94 °C, annealing phase of 30 s at 58–62 °C, and an extension of 1 min at 72 °C. The PCR products were subsequently purified with a TIANgel Midi Purification Kit (Tiangen Biltech Co. Ltd) and sequenced with an ABI 3130XL Genetic Analyzer (Applied Biosystems, Foster City, CA). Sequencing results were assembled and analyzed using Chromas 2.22 software (Technelysium Pty. Ltd., QLD, Australia) with reference sequence in the Universal Mutation Database (UMD).

### Protein structure analysis

A schematic of the cb EGF-like domain of human fibrillin-1 was used to assess the possible impact of the mutation at the secondary structure level [12]. A homology 3D model of the cb EGF-like#15 domain was created based on the Protein data bank (PDB) template 1LMJ [13] (45% sequence identity). DeepView v4.0.1 was used to display the structure file and to predict the potential consequence of the mutation [14].

## Results

### Clinical findings

**Figure 2:**
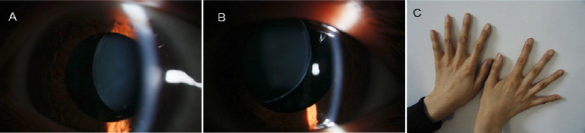
Photographs of the proband. **A** and **B** display the slit lamp photographs of both of the proband’s eyes after the pupils were dilated, showing ectopia lentis. In the right eye (**A**), the lens is dislocated nasally. In the left eye (**B**), the lens is dislocated superonasally. **C** shows arachnodactyly of the proband.

Two members (II:2 and III:12) of the family showed classic MFS (Appendix 2), and the unaffected family members, including a spouse (II:6), appeared normal. The daughter of the proband (IV:4), who was 2 years old, was too young for a clinical diagnosis to be made because several manifestations of MFS are age-dependent and may not yet be present in childhood. The proband had bilateral lens dislocation (Figure 2A,B), high myopia, and strabismus of both eyes. Echocardiography revealed cardiovascular manifestation of dilatation of the aortic root and mild mitral valve prolapse in the proband. Skeletal system abnormalities such as tall stature, long limbs, joint hypermobility, long narrow head, and arachnodactyly (Figure 2C), were also present in the proband. Individual II:2 showed similar clinical symptoms as the proband, with the exception of abnormalities of the cardiovascular system. Lentectomy and intraocular lens implantation were performed on individual II:2 in 2010. Family member II:5 had died of heart disease at the age of 53 and III:2 had died of hepatic cancer at the age of 39, and according to the proband’s description, both of them appeared with tall stature, long limbs, and poor vision.

### Mutation analysis

**Figure 3:**
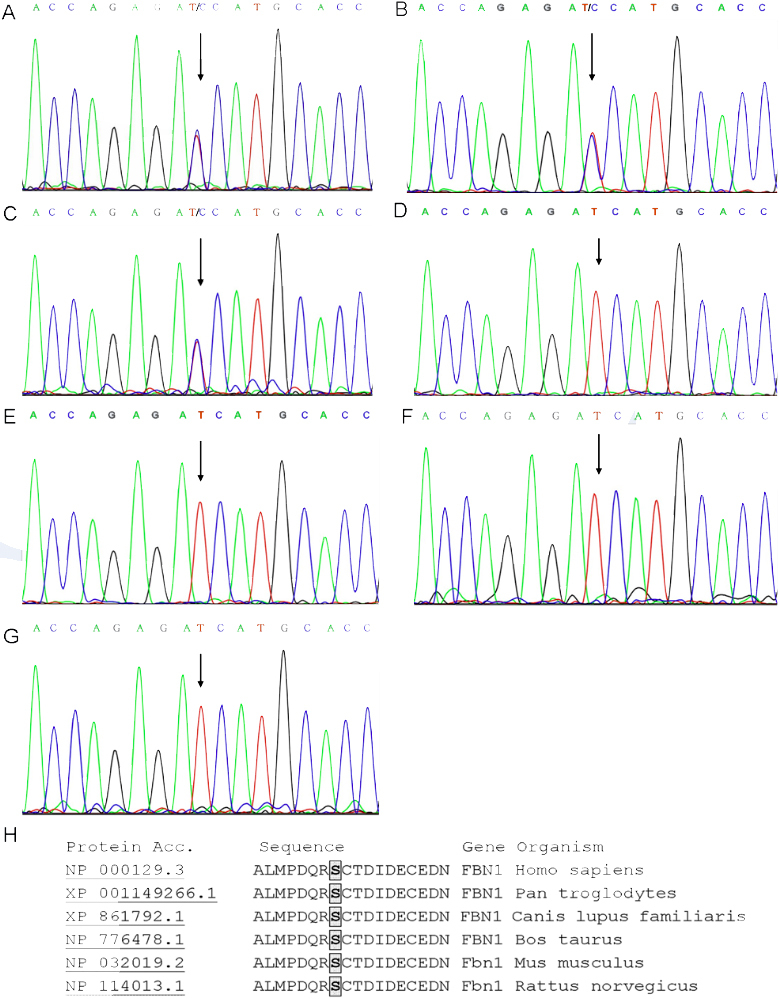
A novel *FBN1* mutation in exon 29. **A**-**C** show a heterozygous T>C transition (indicated by the arrow) resulting in the substitution of serine by proline (S1235P) in the proband, patient II:2, and the proband’s daughter, IV:4, respectively. **D**-**F** show the corresponding normal sequence in unaffected family members II:7 and III:4 and spouse II:6, respectively. **G** shows the corresponding normal sequence in a healthy member. **H** displays the sequence alignment of *FBN1* orthologs surrounding the mutated site using ClustalW. The serine1235 of the human FBN1 protein is highly conserved in several species. These sequences were selected from the NCBI database.

Direct sequencing of *FBN1* revealed a novel heterozygous mutation, c.3703T>C in exon 29, which resulted in the substitution of serine by proline (p.S1235P; Figure 3A). The mutation identified in the proband was also found in patient II:2 and the available affected member IV:4 (Figure 3B,C). No mutation was detected in the healthy family members (Figure 3D-F) or in any of the 170 unrelated control subjects (Figure 3G).

### Potential functional consequences of the mutation

**Figure 4:**
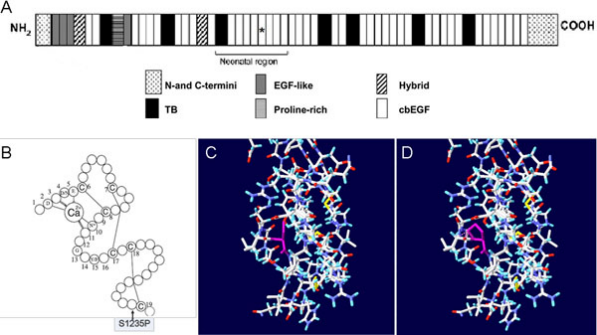
Structure analyses of the missense mutation in the calcium binding (cb) epidermal growth factor (EGF)-like#15 domain. **A** displays the location of the affected module in the neonatal region of the fibrillin-1 domain structure. **B** displays the consensus secondary structure of a prototypical cb EGF-like domain. Calcium binding in the NH_2_-terminal region of the wild-type domain is mediated by the consensus sequence (D/N)-X-(D/N)(E/Q)Xm(D/N)Xn(Y/F) (m and n are variables), and highly conserved amino acids are identified by their single-letter amino acid code. Letter C in the schematic represents the highly conserved cysteines of cb EGF-like domain, and the lines between cysteines represent disulfide bridges. The mutation S1235P is located at the 39th residue of the domain, which neither interferes with calcium binding of the domain nor influences disulfide bond formation. **C** displays the 3D structure of the cb EGF-like#15 domain, which is created based on the Protein data bank (PDB) template 1LMJ (45% sequence identity) by DeepView v4.0.1. The yellow lines represent disulfide bonds, and the purple residue represents the unaffected serine. **D** displays the potential consequence of the mutation. The purple residue represents the substitute proline, and the purple dashed lines mean steric clash with surroundings, which may lead to unstable conformation.

The missense mutation c.3703T>C resulted in the substitution of serine at codon 1235, which is located in the neonatal region of fibrillin-1 (Figure 4A), and is highly conserved among mammalian species (Figure 3H). Secondary structure analysis of the cb EGF-like domain revealed that the mutant proline^1235^ could neither interfere with calcium binding in the NH_2_-terminal region of the domain nor influence disulfide bond formation [15,16] (Figure 4B). Prediction by DeepView v4.0.1 suggested that the mutant residue could probably induce steric clash with its surroundings. Therefore, the conformation of the cb EGF-like domain was likely to be altered by the presence of this mutation (Figure 4C,D).

## Discussion

In this study, we identified a novel heterozygous *FBN1* mutation (p.S1235P) in a four-generation family affected with MFS. This missense mutation is located in the cb EGF-like#15 domain.

It is clear that EGF-like domains play a major role in the pathogenesis of fibrillinopathies, as most of the mutations in these domains are associated with classic MFS [17]. Each cb EGF-like domain of fibrillin-1 contains six highly conserved cysteine residues that form three intra-domain disulphide bonds (C1-C3, C2-C4, C5-C6) and a consensus sequence for calcium binding in the NH_2_-terminal region [15,16]. The mutant residue found in our study, located at the 39th residue of the cb EGF-like#15 domain, did not affect disulphide bond formation or calcium binding of the domain at the secondary structure level. 3D structure analysis by DeepView v4.0.1 showed that the substitution of serine by proline could probably induce steric clash with its surroundings, which probably resulted in a conformation change and had a predictable detrimental effect on its function. It is evident that the structural effects of different *FBN1* missense mutations are complex [18]. Mutations in cb EGF motifs not affecting cysteines or residues of the calcium binding consensus sequence are extremely rare and have been postulated to affect intramolecular packing or intermolecular interactions, and subsequently affect microfiber assembly [13]. Previously, only 6 mutations were published in the UMD website in the cb EGF-like#15 domain, and none of those mutations affected the residues between C-5 and C-6 [7,19-21]. The present study not only suggested that residues between C-5 and C-6 of cb EGF-like domains play an important role in maintaining the function of the FBN1 protein, but also added a new case to the notion that cb EGF-like#14–18 domains (in exons 28–32) are functionally more essential in fibrillin-1 of classic MFS than that of neonatal MFS [22]. This rare mutation found in our study is located in the hot spot region for neonatal MFS and could also cause classic MFS. There is general consensus that neither the location of the affected module in the protein nor the position of the mutant residue is, by itself, sufficient to predict potential genotype-phenotype correlations [23].

This missense mutation was also found in the proband’s daughter, who was 2 years old and did not yet fulfill the diagnostic criteria. As such, in situations of clinical uncertainty, molecular analysis of *FBN1* seems a logical aid in the clinical diagnosis of MFS [18]. Moreover, the most important complication of MFS is a progressive dilatation of the aortic root and ascending aorta, leading to aortic valve incompetence and aortic dissection [24]. As a result, early recognition of at-risk individuals not (yet) fulfilling the diagnostic criteria by molecular diagnosis is important in view of the available treatments that can significantly improve life expectancy and also offer the possibility of prenatal diagnosis.

Although the four-generation MFS family we studied was large, the number of patients was relatively small. For genetic studies, it is better to examine pedigrees with more affected individuals, because (1) to find the mutant gene and exclude genetic heterogeneity, linkage analysis for candidate gene screening should be initially done, and in this condition, numbers of affected individuals should be large enough; and (2) more patients can help investigators to better interpret the rule of inheritance of the disease and display the genotype-phenotype correlations.

Collectively, evidences from our study and published data supported that this novel p.S1235P mutation is the causative mutation for MFS in this four-generation family. Mutations in cb EGF-like domains of FBN1 play a critical role in the pathogenesis of classic MFS, and molecular diagnosis of MFS is becoming more and more important. Although more than 600 *FBN1* mutations have been published in the UMD website for MFS, information on genotype–phenotype correlations is still limited, especially in the Chinese population. Our data adds a novel mutation to the spectrum of *FBN1* gene mutations, analyzes the potential functional consequence of the mutation, and enriches the existing knowledge of genotype–phenotype correlations of Marfan syndrome.

## Appendix

Appendix 1. Primers used for *FBN1* amplification. Summary of the primers used for the amplification of *FBN1* exons. Sequences are given in the 5′→3′direction. To access the data, click or select the words “Appendix 1.” This will initiate the download of a compressed (pdf) archive that contains the file.

Appendix 2. Clinical details of the patients II:2, III:12, and family member IV:4. Each patient in the family has bilateral ectopia lentis, myopia, arachnodactyly, and joint hypermobility. Only the proband displays abnormalities of the cardiovascular system. Individual IV:4 appears normal. Abbreviations: M: male; F: female; IOL: intraocular lens; AS/H: arm span/height ratio; +: present; –: absent. N: normal. To access the data, click or select the words “Appendix 2.” This will initiate the download of a compressed (pdf) archive that contains the file.
